# Understanding stakeholder views of the use of digital therapeutic interventions within children and young people’s mental health services

**DOI:** 10.3389/fpsyt.2025.1505345

**Published:** 2025-02-11

**Authors:** Brioney Gee, Tim Clarke, Jess Garner, Bonnie Teague, Georgianna Coote, Aoife Dunne, Rachel McGuire, Andrew Laphan, Manjul Rathee, Jon Wilson

**Affiliations:** ^1^ Norfolk and Suffolk NHS Foundation Trust, Hellesdon Hospital, Norwich, United Kingdom; ^2^ Norwich Medical School, University of East Anglia, Norwich, United Kingdom; ^3^ Unit C, Health Innovation East, Cambridge, United Kingdom; ^4^ Independent Researcher, Manchester, United Kingdom; ^5^ BFB Labs, Shift Design, London, United Kingdom

**Keywords:** mental health, digital health technologies, digital therapeutics, children, young people, implementation

## Abstract

**Background:**

Providing the growing number of children and young people seeking mental health support with timely access to care poses a significant challenge. Increased use of digital technology in the delivery of children and young people’s mental health services has been proposed as a means of increasing access to treatment.

**Methods:**

We conducted three interrelated studies to provide multi-perspective insights into the use of digital therapeutic interventions within children and young people’s mental health services in the UK. Study 1 used semi-structured interviews and an online survey to collect the views of digital therapeutic interventions of families who self-identified as facing additional barriers to accessing mental health support (n=13). Study 2 involved eight focus groups with children and young people’s clinicians, service managers, commissioners, and policy leads (n=28), exploring participants’ views and experiences of implementing and sustaining digital therapeutic interventions. Study 3 was a consensus exercise which aimed to identify actions needed to bridge the gap between the development and use of digital therapeutic interventions for children and young people’s mental health through focus groups with parents/carers and professionals (n=17), and three Delphi-survey rounds.

**Results:**

Our findings revealed considerable enthusiasm for the increased use of digital mental health interventions for children and young people across stakeholder groups, but also identified key barriers to their implementation. Actions perceived to facilitate more effective implementation included: a) co-producing interventions, commissioning decisions and implementation plans with children and parents/carers, b) enhancing national guidance and local leadership, c) integration of digital offers within existing clinical pathways, and d) efforts to ensure accessibility and inclusivity.

**Discussion:**

Digital therapeutic interventions offer a promising solution to the challenge of improving access to mental health support for children and young people. Strengthened guidance and leadership, sustained funding and further evidence-generation are urgently needed to enable this promise to be realised.

## 1 Introduction

Digital technologies are playing a growing role in the delivery of mental health services, facilitating novel approaches to intervention. It has been suggested that this digital transformation is likely to be the most impactful in the case of mental health services for children and young people (1, 2). This view reflects the currently poor access to treatment for children and young people experiencing mental ill-health (3), as well as high levels of digital literacy among young people who have grown up with digital technology as a ubiquitous aspect of everyday life (4).

Digital therapeutic interventions are defined as evidence-based interventions that use software applications to prevent, manage and treat health conditions (5). They can be used as standalone treatments, or in conjunction with medication or other therapies. Digital therapeutics fall within the broader landscape of digital health technologies, which includes telehealth, digital health devices and wearables, electronic health record systems, and consumer health apps. Digital therapeutic interventions have been found to be effective in reducing symptoms of depression and anxiety in young people in comparison to non-active control conditions (6, 7).

Since the majority of digital therapeutic interventions for young people’s mental health require minimal therapist support (6), these interventions have the potential to be highly scalable. Given the limited capacity of specialist child and adolescent mental health services and consequently high access thresholds and long waiting times (8), growing the use of digital therapeutic interventions holds promise as a means of increasing timely access to evidence-based support. Digital therapeutic interventions also have the potential to overcome attitudinal barriers that may prevent young people from seeking support (9, 10). Further, digital therapeutic interventions may contribute to reducing health inequalities through making access to evidence-based treatment more equitable (11).

Despite the potential benefits of digital therapeutic interventions, adoption of these technologies as part of routine service provision has proved slow (2). A recent systematic review found that less than half of app-based mental health interventions found to be efficacious in research settings had been successfully adopted and sustained in the real-world (10). Where interventions have been implemented in routine care, poor uptake, engagement and adherence have often been concerns (12). It has been reported that scepticism among clinicians, parents/carers and young people about whether digital interventions offer the same level of benefit as face-to-face treatment may contribute to suboptimal implementation (13).

The widely applied Technology Acceptance Model (14) suggests that the adoption of new technologies is influenced by the perceived usefulness and perceived ease of use of the technology. The model proposes that stakeholder views regarding usefulness and ease of use are determined both by individual differences and external factors such as social influence. Successful adoption and sustainment of digital therapeutic interventions within children and young people’s mental health services will be dependent on the views of a range of different stakeholders. These stakeholders include young people and their families, healthcare professionals, service managers and commissioners, policy leads, academics and technology developers.

The current paper presents the findings of three interrelated studies completed as part of the I-DIGIT (**I**nvestigating **Digi**tal **T**herapy) project. I-DIGIT aimed to provide insights into the use of digital therapeutic interventions within UK NHS-funded children and young people’s mental health services.

Study 1 aimed to explore the views of digital therapeutic interventions of young people and their parents/carers, with a particular focus on families who face additional barriers to accessing mental health support. Study 2 aimed to understand the views of healthcare professionals, service managers and commissioners on how digital therapeutic interventions can best be implemented and sustained within NHS-funded children and young people’s mental health services in England. Study 3 aimed to achieve a consensus (across mental health professionals, commissioners, academics, policy leads, industry experts and parent/carers) on the actions needed to bridge the gap between development and implementation of digital therapeutic interventions for children and young people’s mental health. The findings of these studies were considered together to generate key recommendations to maximise the likelihood of the transformative potential of digital therapeutic interventions for children and young people’s mental health being realised.

## 2 Methods

### 2.1 Study 1

#### 2.1.1 Design

Semi-structured interviews and an online survey were used to collect qualitative data on the views and experiences of digital therapeutic interventions of children with experience of mental health difficulties and their parents/carers. The interview topic guide was developed in collaboration with our young advisors, who role-played the interview using an initial draft of the topic guide and provided feedback before it was finalised. Topics covered included perceptions of digital therapeutic interventions as a treatment option, how digital therapeutic interventions should be used in practice, what factors support engagement with digital interventions, and barriers to engagement with these interventions.

The online survey covered the same topics included in the interview topic guides, but with adaptations to allow for an asynchronous written format. The option to respond via online survey was added as an amendment to the study to broaden the range of views we were able to collect. This was in response to difficulties with recruitment and feedback that some young people experiencing anxiety and with additional barriers to accessing support did not feel comfortable participating in an interview, but would nevertheless value the opportunity to contribute their views. Once this option was introduced, participants had the option to participate either by taking part in the interview or by completing the survey according to their needs and preferences.

#### 2.1.2 Participants

Eligible families had at least one child aged 7-12 years old and self-identified as facing additional barriers to accessing mental health support (including, but not limited to, difficulties accessing support related to socioeconomic factors, race, ethnicity or culture, disability, or neurodiversity). The study was promoted across participating clinical services, parent groups and on social media. In total, 13 young people and their families participated, four (two girls, two boys) via a semi-structured interview, and nine (five girls, four boys) via the online survey.

#### 2.1.3 Procedures

Semi-structured interviews with young people took place in participants’ homes in accordance with the preferences of the families. At least one parent/carer or carer was present throughout the interview. The researcher used the topic guide flexibly, adjusting language, play materials and pictorial support to engage children and meet specific communication needs. Interview durations ranged from 15-35 minutes. Interviews were audio recorded and transcribed verbatim.

The survey questions covered the same topics as the interview topic guides, adapted for administration in written format, and was designed for parents and children to complete the questions together. The study was promoted by participating clinical services in the East of England, through local parent/carer groups and support charities, in public venues frequently used by families, and on social media.

#### 2.1.4 Analysis

Interview transcripts and survey responses were analysed using deductive content analysis (15) to explore the difference in views between participants and accommodate the different methods of data collection. Interview transcripts and survey responses were coded by authors BT and AL based on key words and phrases used by children and parents, and umbrella terms generated to describe a similar response or view. These key words were then grouped in broader themes, working on interview transcripts and survey responses individually initially, before triangulating across the two data types.

### 2.2 Study 2

#### 2.2.1 Design

Online focus groups were conducted with children and young people’s mental health clinicians, service managers and commissioners to collect qualitative data on professional’s views and experiences of implementing and sustaining digital therapeutic interventions within NHS-funded mental health services for children and young people.

#### 2.2.2 Participants

To be included in the study, participants had to be employed as a children and young people’s mental health clinician, service manager, commissioner or policy lead. Participants were recruited through a combination of purposive (aiming to achieve diversity of professional background, service type and geography), opportunistic, and snowballing sampling. The research team made efforts to contact all NHS mental health trusts in England to request support with promotion of the study and to provide trusts with the opportunity to opt-out of allowing their staff to participate. Of 54 organisations approached, seven opted-out. In total, 28 participants employed by 17 different services across England took part in Study 2. Nineteen participants were employed by NHS organisations, five in a voluntary, community or social enterprise organisation, and four by a local authority. Fifteen were clinicians, eight service managers, three commissioners, and two policy leads.

#### 2.2.3 Procedures

Eight focus groups were conducted. All were held online using Microsoft Teams and each lasted approximately 90 minutes. Two facilitators were present at each group: author TC who chaired the discussion supported by AD or AL. The size and composition of each group was determined by participant availability. The focus group topic guide was developed based on the Consolidated Framework for Implementation Research (16) to facilitate exploration of specific implementation factors relevant to the adoption and sustainment of digital therapeutic interventions. Topics explored included participants experiences of implementing digital therapeutic interventions and the barriers to, and facilitators of, digital interventions being implemented within children and young people’s mental health services. The focus groups were recorded and transcribed verbatim.

#### 2.2.4 Analysis

Transcripts were analysed thematically following the procedure described by Braun & Clarke (17). First, each transcript was read and coded by at least two members of the analysis team (TC, AD, BG, AL, RM, JW), the team then met to discuss this coding, resolve any discrepancies through consensus discussions, and develop initial themes. These initial themes were then reviewed against the data by individual team members, before meeting again to refine and agree the final set of themes.

### 2.3 Study 3

#### 2.3.1 Design

A Delphi-informed consensus exercise was conducted with the aim of achieving agreement across stakeholder groups on actions needed to bridge the gap between the development and use of digital therapeutic interventions for children and young people’s mental health. The consensus exercise involved online focus groups with professionals working to develop digital health technologies for children and young people’s mental health and parents/carers with an interest in this topic. Insights from these focus groups were combined with findings of Study 2 to generate a bank of items which were prioritised by a panel of experts comprising mental health professionals, commissioners, academics, policy leads, industry experts and parent/carers through a series of three online survey rounds.

#### 2.3.2 Participants

To be eligible to take part in the online focus groups, participants had to either be a parent or carer with experience of supporting a child experiencing mental health difficulties, a professional working to develop digital technologies for children and young people’s mental health services or academic conducting research on this topic. Three focus groups were conducted involving a total of 17 participants: six parents/carers and 11 professionals.

Individuals invited to form the Delphi panel were recruited via the research team’s existing networks, including those who took part in Study 2 and the online focus groups for this study. Potential panel members were invited to take part by email, which included a copy of the information sheet and consent form, and a link to complete the consent form and survey items should they wish to take part. A total of 88 people were invited to participate, including mental health professionals, commissioners, academics, policy leads, industry experts and parent/carers.

#### 2.3.3 Procedures

Three focus groups were conducted: two with professionals and one with parents/carers. All groups were held online using Microsoft Teams and lasted approximately 90 minutes. Two facilitators were present at each group. The topic guides for the discussions explored the process of developing digital therapeutic interventions for children and young people’s mental health, experiences of adoption of digital therapeutic interventions by the NHS, the standard of evidence required for adoption, and practical steps that could be taken to help bridge the gap between development and uptake. The focus groups were recorded and transcribed verbatim.

The findings of the focus groups were combined with information collected in Study 2 to create a bank of items to be ranked by the panel through a series of surveys, with the aim of facilitating the convergence of opinions into a consensus view. Each of the three survey rounds included a list of statements with which the panel was asked to indicate their level of agreement. In the first two rounds, panel members were asked to rate their agreement using a Likert scale of 0 (strongly disagree) to 10 (strongly agree). A ‘not relevant’ option for each statement was included where a panel member did not feel able to rate a particular statement, as well as a final free text box to capture additional statements suggested by the panel. First round statements that were strongly agreed with (70% or more rating it ‘7’ or above) advanced to the second survey round. Second round statements that were strongly agreed with (70% or more rating it ‘8’ or above) advanced to the third survey round. There is not currently an established best practice for defining consensus in a Delphi exercise, but the criteria and thresholds selected were in line with other similar studies (18). For the third and final survey round, the panel were asked to rank statements in order of importance.

#### 2.3.4 Analysis

Transcripts of the focus groups were analysed thematically using a framework approach (19) as this is well suited to collaborative analysis by multi-disciplinary teams. The analysis team comprised JG, GC, AD and RM). Framework analysis is a structured analytic approach comprising six stages: data familiarisation, initial coding, developing an analytic framework, applying the analytic framework, charting data into a matrix, and interpretation. Following completion of this analysis, the resulting themes were used in conjunction with the findings of Study 2 to develop the bank of statements to include in the consensus survey. Analysis of survey results involved calculating the proportion of participants in each round who rated or ranked each statement above the threshold for progression to the next round or for inclusion in the final list of consensus statements.

### 2.4 Ethics

Ethical approval was obtained from the NHS Health Research Authority (HRA) following a favourable ethical opinion from North West – Preston Research Ethics Committee (REF:22/NW/0195). All participants provided informed consent (or assent and parental consent for young people aged under 16 years), recorded either via completion of a written consent form or audio-recorded verbal consent. Those participating in a personal capacity (young people and parents/carers) received shopping vouchers at a rate of £10 per hour to thank them for their time and were reimbursed for any expenses. Those participating as part of their professional role did not receive compensation for their time over and above their usual salary.

### 2.5 Patient and public involvement

We conducted all three studies alongside a panel of young advisors who gave advice on participant recruitment, contributed to the interpretation of study findings, and supported dissemination efforts. In total, 13 young people ranging from 8 to 24 years old were involved in the project as young advisors. Young advisors were paid for their time and offered opportunities to take part in training to build their research skills, as well as letters of recommendation/references to support their educational or professional development.

RM (a peer researcher with expertise in the experiences of parents/carers supporting young people with mental health difficulties) joined the research team to support the delivery of Study 3. She contributed to recruitment of parents and carers, co-facilitated the parent/carer focus group, and contributed to analysis and interpretation of qualitative data.

## 3 Results

### 3.1 Study 1

Key findings of the study were organised into three themes reflecting the views of digital interventions of families who self-identified as facing additional barriers to accessing mental health support. These were: *‘Enthusiasm for digital approaches’, ‘Balancing engagement and effectiveness’, and ‘Need for a flexible approach to delivery’.*

#### 3.1.1 Enthusiasm for digital approaches

All participating young people and parents/carers expressed enthusiasm for the idea of digital therapeutic interventions, however none of the young people interviewed, and only one of the survey respondents, had previously been offered a digital intervention as part of their mental health treatment. Participants perceived the key benefits of digital interventions to be more immediate access to support, more fun and the flexibility to access the intervention at a time convenient to them, with no travel required.

Parents and carers of children with multiple complex health needs (n=3) expressed a particular enthusiasm for digital therapeutic interventions. They expressed that their children frequently fall between gaps in formal service provision and viewed being able to access digital interventions as a potential means of overcoming this challenge. One parent stated:

“Accessing help has been really difficult, so to have something you can easily access fills that gap”

Young participants expressed that they believed digital therapeutic interventions might help them *“feel normal”*, *“be relaxed”*, *“be happy”* and *“release feelings of stress and worry”*.

#### 3.1.2 Balancing engagement and effectiveness

All young participants enjoyed playing digital games recreationally, and shared that their favourite games were fun, had clear goals and a sense of progress. Digital therapeutic interventions with a gamified interface mirroring the features of games they already play were seen as most appealing, such as being able to select their own avatar or character. Parents expressed some conflict regarding the idea of supporting their child to engage in digital interventions whilst simultaneously also trying to limit their recreational screen time. However, they felt that the additional screen time would be worthwhile if it improved their child’s mental health. One parent commented:

“[my child] does struggle to get away from video games … [but] if it was helping her anxiety, then I would be fine with her playing it whenever she felt that”

Some parents expressed uncertainty regarding the effectiveness of digital interventions, but another felt that they may be preferrable to face-to-face support since this had not proved helpful for their child. Parents shared that to support their child to sustainably use the intervention, they would need information about what meaningful changes they could observe in their child within the home environment, beyond being simply informed that the interventions were effective.

#### 3.1.3 Need for a flexible approach to delivery

There were differences between family members in how they preferred digital therapeutic interventions to be delivered. Children wanted to have digital therapies available on demand for when they felt they needed to use them. One young interviewee expressed that their current mood would impact their motivation to engage with a digital intervention:

“if you are really happy and there’s like nothing you want to change, then just leave your life as it is. If you’re really sad, that’s when you can see what you can improve”

Another stated:

“some days like Monday, let’s say, I have a really bad day and then I want to use it that day”

However, some parents and carers preferred a more structured approach, with guidance on when and how their child should use the intervention being provided to families as part of the digital offer. They expressed concern that without this guidance and clear information about the intended effects of the digital intervention, they would be unable to support their child to engage with the intervention to achieve the most benefit.

There was a diversity of opinions on where digital interventions should be offered. Some families felt that schools would be an appropriate and convenient venue to offer support. However, one child felt it was important to separate support from school, and would find it off-putting if the intervention felt too educational offered in that setting. Another young person expressed that they would be more likely to engage with an intervention that was recommended to them by a healthcare professional, and two stated that they would prefer to use digital therapeutic interventions alongside seeing a healthcare professional in-person, rather than as a stand-alone offer.

Very few barriers to the use of digital therapeutic interventions were identified by families. Two families identified only having access to one type of device (laptop or phone) as a potential barrier if the digital intervention was not available across all devices, and one child expressed that lack of privacy in the home might make it difficult to engage with a digital intervention.

### 3.2 Study 2

Four themes (three with subthemes) were developed in relation to participants’ (children and young people’s mental health clinicians, service managers, commissioners and policy leads) views of the implementation of digital therapeutic interventions within children and young people’s mental health services. These themes were named: *‘Digital perceived to be second best’* (Subthemes: *‘(Dis)integration into clinical pathways’* and *‘Workforce culture’*); *‘Need for strengthened leadership and guidance’* (Subthemes: *‘Navigating the evidence’* and *‘Digital leadership’*); *‘Need for sustainable funding’*; and *‘Equality of accessibility’* (Subtheme: *‘Co-production’*).

#### 3.2.1 Digital perceived to be second best

While most participants expressed positive views about digital therapeutic interventions, the perception that digital interventions are second best to traditional face-to-face interventions was widespread. Professionals described feeling that they were *“fobbing off”* service users if they offered them a digital intervention rather than a face-to-face treatment. This seemed to reflect the perception that most families seeking mental health support expect and prefer in-person treatment.

**“**I think they want the real deal, they want, they want to see the clinician and if they’re giving them an app, that’s not what they think they need, they want. They, they want that, they want that treatment, they want to see that person.’

Some participants reported having experienced offering families a digital intervention and receiving feedback that they felt *“sold short”* by not being offered a face-to-face appointment with a clinician.

##### 3.2.1.1 Subtheme: (dis)integration into clinical pathways

Reflecting the perception of digital therapeutic interventions as ‘second best’, most participants viewed digital interventions as being most suitable as waiting list interventions, early help offers or as an adjunct to other interventions, as opposed to standalone clinical pathways. However, most felt that digital interventions were not yet sufficiently embedded within clinical pathways.

“I think they’re at ad hoc or, or kind of they’re separate, they’re, they’re something that we’re signposting to as opposed to being integrated within our care.”

A need for consensus regarding the place of digital interventions within clinical pathways and how best to ensure their integration with other treatment offers was apparent.

##### 3.2.1.2 Subtheme: workforce culture

It was suggested that workforce culture may prove a barrier to implementation of digital therapeutic interventions within services. Participants perceived that enthusiasm for digital innovation among staff members was mixed, with some feeling a degree of reticence about their use.

“Some practitioners have really been enthusiastic and engaged with the whole digital offer, when it came in, and others have been just very resistant actually. “Look, this just doesn’t work”. And then you’ve got others in the middle who say this is good enough in the circumstances.”

Some participants speculated that the source of this resistance could be fear of their current job role being made obsolete, or anxiety about being asked to use new technologies due to a low level of professional digital literacy.

#### 3.2.2 Need for strengthened leadership and guidance

Participants articulated a need for clearer guidance regarding which digital therapeutic intervention they should be commissioning and adopting, as well as a need for strengthened leadership to support the implementation and sustainment of recommended interventions.

##### 3.2.2.1 Subtheme: navigating the evidence

There was a consensus on the need for evidence-informed, regularly updated guidance to inform commissioning and implementation of digital therapeutic intervention. Most participants told us that they believed it was important that all interventions that are used within children and young people’s mental health services are evidence based. However, most were unclear about whether the interventions they were commissioning or using within their services were supported by evidence, and the standard of evidence that would be acceptable.

A minority of participants reported that they had attempted to explore the evidence-base of interventions they were considering implementing. Useful resources mentioned included those provided by the Organisation for the Review of Care and Health Apps (ORCHA), NHS websites and the (now decommissioned) NHS Apps Library. It was suggested that there was an unmet need for a well-know, centrally maintained and updated resource listing approved digital interventions for use within NHS-funded mental health services.

“That would be really useful, wouldn’t it? To have a sort of resources database that we all know about, and we can go and check these things out that somebody had checked out the, the usefulness”

##### 3.2.2.2 Subtheme: digital leadership

Many participants felt there is a need for services to have dedicated digital leads whose responsibility it would be to appraise, select, implement and support the use of digital therapeutic interventions. It was suggested that, in the context of overstretched services in which clinicians must balance multiple priorities, it is likely that initiatives to implement digital interventions will fail without a staff member(s) whose specific job role it is to facilitate this implementation. Reflecting on a recent initiative, one participant said:

“we realised that digital technologies really needed to be held within its, within its own right rather than trying to muddle along like we often do … I was quite clear that it needed to be somebody that had dedicated time to think about creating the [digital technology] strategy”.

However, participants also expressed that it is important that the use of digital interventions is supported throughout an organisations’ management structure, including by senior leadership. Further, building and maintaining good relationships between technology developers, policy makers, commissioners, and healthcare staff was seen as essential at all stages of development and implementation.

#### 3.2.3 Importance of sustainable funding

Participants viewed the potential cost-effectiveness of digital therapeutic interventions as a key benefit and believed that this should be evaluated to inform intervention selection. However, concern was expressed about a lack of long-term, ring-fenced funding for digital interventions, money instead being ‘syphoned’ from existing budgets or allocated on a short-term basis when there is underspend in other areas. Commissioners highlighted that most digital therapeutic interventions do not currently count towards access targets and often provide limited outcome data. This makes it difficult to establish the impact of digital interventions and therefore to justify their prioritisation.

“[investing in] digital is a really hard thing to be able to justify when, when from the digital you’re not getting the outcome measurement tool that you might get from being with somebody”.

Several participants felt strongly that families should not incur any personal cost to access digital therapeutic interventions so would not feel comfortable signposting to any intervention that must be paid for by the service-user. It was also highlighted that where funded access to a digital intervention would be time-limited, this should be made clear to the family from the outset.

#### 3.2.4 Equality of accessibility

Participants expressed that digital therapeutic interventions might play a role in improving access to treatment, particularly for young people who face additional barriers to accessing non-digital services. For instance, several participants highlighted the benefits of digital therapeutic interventions being accessible from anywhere for families who would struggle to attend a clinic appointment.

“We also have a, a sort of a group of families in particular areas that are socioeconomically very deprived. And so, you know, paying £15 to get bus fare to come to the clinic isn’t always possible. So to be able to have that alternative option is so helpful on a practical level.”

Digital therapeutic interventions were also seen as potentially making treatment more accessible to some young people with specific communication or sensory needs, for instance neurodivergent young people.

However, participants were also mindful of the potential for digital therapeutic interventions to exclude some young people. Many participants shared concerns about the impact of digital poverty on equitable access to digital therapeutic interventions. Participants articulated that some young people most in need for mental health support, including those living in socioeconomically disadvantaged or rural areas, may not be able to access digital interventions due to unreliable internet access, not being able to afford data or not having use of a suitable device.

“we have to be realistic that quite often a lot of the higher needs is in a particular part of young people who might not be able to access a device to do a digital intervention or they might [not] even have access to Wi-Fi”

Participants also spoke about the value of clinic appointments for young people who lack privacy in their home environment or who do not have stable housing.

“I work with a lot of older young people so you know trouble with homelessness and, and that kind of thing. And actually, coming to us is the only warm hour of their week.”

For younger children, the availability of a parent or carer to help set-up and supervise the use of a digital therapeutic intervention was identified as a barrier to access faced by some children. Further, whilst digital interventions were seen as helpful in meeting the needs and preferences of some neurodiverse young people, participants also highlighted that others might struggle to engage with a digital intervention without adaptations or additional support. As such, participants felt it important that digital therapeutic interventions are implemented as part of a menu of treatment options, rather than being assumed to meet the needs of all young people.

##### 3.2.4.1 Subtheme: ‘co-production’

There was agreement among participants that involving young people and their families in the development, commissioning, and implementation of digital therapeutic interventions is important to optimising their potential benefits. Participants expressed the belief that co-production increases the likelihood of digital interventions meeting the needs of as many young people as possible and being sufficiently appealing to sustain engagement.

“you could have the most fantastic system, but if a young person doesn’t find it appealing to actually perhaps log on and use, it’s not going to be any value in it at all.”

However, while there was agreement regarding its importance, few participants were confident that co-production was occurring, or had experienced co-producing the commissioning or implementation of digital technologies.

### 3.3 Study 3

#### 3.3.1 Focus group findings

The initial focus groups with a subset of panel members, both professionals and parents/carers, raised a wide range of issues related to bridging the gap between development and uptake of digital therapeutic interventions for children and young people’s mental health. These can be characterised as falling within one of six themes: *‘Importance of alignment with existing service structures’*, *‘Poorly defined routes to implementation’*, *‘Need for novel approaches to evidence generation’*, *‘Need to create a digitally skilled workforce’* and *‘Importance of co-production’*.

##### 3.3.1.1 Importance of alignment with existing service structures

Alignment with NHS delivery structures, services and pathways was seen as a valuable enabler of uptake of digital therapeutic interventions. Professional participants described the process of understanding what the NHS delivers and where digital interventions can offer a solution to existing challenges as essential to successful implementation and sustainment.

“Your value proposition and the evidence has to demonstrate the value against those pain points, so I know this all sounds very kind of blindingly obvious, but I think sometimes it gets missed in the complexity that can be NHS commissioning”

Professional participants believed it would be beneficial for mental health services to work directly with technology developers to understand how a proposed innovation would fit with service delivery. This was viewed as a preferential approach to either a commissioner or senior NHS leader imposing a technological solution, or a technology developer working in isolation. Professional participants recognised a need to come together early in the development process to ensure alignment with service needs and processes, as well as interoperability with existing digital systems and devices. There was agreement that implementation of digital innovations would be significantly easier if there was more consistency across NHS services, for example common children and young people’s mental health care pathways, shared digital infrastructure and consistent expectations regarding outcome reporting.

Parents and carers were enthusiastic about digital therapeutic interventions being offered by NHS funded mental health services. They felt that this mode of delivery would suit many young people given their familiarity and comfort with digital technology.

“It’s all they know, isn’t it? It’s, it’s all children know, technology is their world, so I think it just needs to be embraced”.

However, supporting the need for digital interventions to be integrated within existing service structures, parents and carers felt that it was important that services continue to offer families choice in how their child is supported. Parents emphasised that digital therapeutic interventions will not be a suitable treatment option for all young people.unfair

##### 3.3.1.2 Poorly defined routes to implementation

Professional participants identified a lack of clearly defined routes to implementation of digital interventions within NHS-funded children and young people’s mental health services as an important barrier. Technology developers experienced the approvals process for NHS adoption of a digital therapeutic interventions as inconsistent, and found it was often unclear who is responsible for approval decisions. Participants felt that implementation would be facilitated by services following a consistent national approval and commissioning process rather than idiosyncratic local decision-making processes. However, existing tools to assess the suitability of digital interventions for use with NHS services, such as the Digital Technology Assessment Criteria (DTAC), were perceived to be not well-known and poorly understood by NHS decision makers.

Participants identified that very limited guidance is available to support technology developers to navigate complex systems in order for a novel technology to be adopted within NHS services.

“I think then having enough capital to be able to have the time to navigate this complex purchasing, the fragmented system that is the NHS, is actually one of the critical success factors. If I had my time again I wouldn’t start in the UK because it takes a long time to become established”.

Similar issues were identified in relation to an absence of mechanisms to support implementation of digital therapeutic technologies developed through academic research and development processes.

*“There are so many things being developed within universities, funded mostly by NIHR [National Institute for Health and Social Care Research], developing new interventions that are incredibly, you know, high-quality based on amazing clinical input. You know based on great evidence behind them. And then there’s no platforms take them forward to the NHS. And so they just sort of sit, you know sit there getting dusty on a shelf and never really get used”.*

Parents and carers felt it important that approval processes ensure that the safeguarding implications of a digital therapeutic intervention have been appropriately addressed, and that there is a mechanism for obtaining parental consent before a child accesses a digital intervention.

##### 3.3.1.3 Need for novel approaches to evidence generation

Professionals expressed concern that traditional academic approaches to establishing intervention effectiveness, in particular randomised control trials, can add years to the development process. This was seen as problematic in a fast-changing technological landscape as it delays the adoption of promising new technologies and prohibits iterative adaptations to improve digital interventions based on user feedback.

“In trials you can’t keep adapting the intervention, which is problematic … I think there’s a lot that we can learn from industry partners about how to get things up and running”.

“There’s competition too. If you spend, you know, three years doing an RCT [randomised controlled trial] for your particular product, there could be five, you know, new products that just use your RCT as evidence that of course it’s effective”.

There was agreement that digital therapeutic interventions require new and innovative research and evaluation methods to demonstrate their value. Real-world evaluations, demonstrating how an innovation works in the environments in which staff and users would be adopting it, were seen as valuable. Some participants asserted that this type of evaluation to show clinical value should take place prior to marketing and commercialisation of a product.

“You need to conduct some real-world value research with clinicians and patients involved to ensure it has value to the health system and the patient. We did that real-world evaluation at a low cost and it set the base to think about what our proposition was to all the different stakeholders that we have”.

Parents and carers tended to rely on the lived experiences of other parents or carers to assess the likely effectiveness of a digital therapeutic intervention for their child.

“As a mum … parent reviews are just the things that I go by most of the time. If they’re lacking, I don’t even entertain a company, really, I want to know what parents have said … I always go to Trustpilot, but I like to, I like to find people in Facebook groups and I like to find the real person and the real deal”.

##### 3.3.1.4 Need to create a digitally skilled workforce

Professional participants identified a need for additional tools and training to enable the children and young people’s mental health workforce to make informed decisions about commissioning, implementing and using digital therapeutic interventions. There was a perception that many commissioners, clinicians and service managers need additional support to understand the depth and breadth of digital technologies available and how to distinguish between them.

Dedicated resource to support implementation was viewed as important to effective uptake and usage. Participants highlighted that, in some cases, technology developers offer their own team members to support implementation and, in others, local systems had assigned specific staff members to perform this role.

“In Manchester they are rolling out the idea of ‘digital navigators in healthcare’, people who have expertise and understanding of how to use these digital interventions, and it comes with a package of care delivery”.

There was support for the idea of appointing “digital champions” within services to provide other staff members with training and support to implement digital innovations.

##### 3.3.1.5 Importance of co-production

Participants agreed the importance of involving young people and parents/carers in the development and implementation of digital therapeutic interventions. It was felt that further work was needed to facilitate co-production with families, particularly those from demographic groups who are less often heard. While examples of good practice in involving end users in the design of digital interventions were identified, co-production practice was perceived to be inconsistent, with a lack of clear standards.

#### 3.3.2 Survey rounds

The findings of the focus groups were combined with information collected in Study 2 to create a bank of 45 statements. The full list of statements is available as an online supplement to this article. In total, 32 participants responded to the first survey round. **Table 1** summarises the professional sectors participants indicated they worked within**/**whether they were responding as a parent/carer representative.

**Table 1:** Round 1 respondents by professional sector or parent/carer status.

Respondent type	N
NHS	11
University/Higher Education	5
Parent/carer	3
National body	3
Voluntary sector	3
Local authority	2
Private healthcare/technology provider	2
Technology development company	2
Local authority/ICB (Integrated Care Board)	1

A total of 22 of the 45 statements met the prespecified consensus threshold for progressing to the next survey round (70% or more of the panel scoring the item ‘7’ or above on the 0-10 Likert scale). In addition, eleven free text responses were received. Additional statements suggested were reviewed for overlap with the 22 statements that met the threshold for inclusion, and six further statements were added to the next survey round. As such, a total of 28 statements were included in the second survey round (see **Supplementary Material**).

The second survey round received 30 responses (27 professionals, 3 parent/carers). A higher consensus threshold of 70% of the panel scoring a statement ‘8’ or above was used for this round as 27 of 28 statements were rated ‘7’ or above by 70% of the panel. Seventeen statements met the increased consensus threshold and were progressed to the final survey round (see **Supplementary Material**). Free text box responses were reviewed but no additional statements were added as all suggestions were accounted for by the already included statements.

In the third survey round, the panel were asked to rank the final 17 statements in order of importance. Twenty-two panel members completed the third survey round (21 professionals, 1 parent/carer). The percentage of respondents who ranked each statement within their top five most important was totalled to produce a final consensus ranking. **Table 2** shows the eight statements ranked most important by the panel.

**Table 2:** Top ranked statements in final survey round ordered by percentage of respondents who ranked each statement within their top five priorities.

Statement	%
When developing digital health technologies for children and young people’s mental health, a panel of young advisors should be consulted to inform development.	72.7
A national strategy for digital health technologies for children and young people’s mental health needs to be established and made publicly available.	45.5
When developing digital health technologies for children and young people’s mental health a panel of parents and carers should be consulted to inform development.	40.9
Digital health technologies should be integrated with existing pathways for children and young people’s mental health.	40.9
There should be a National Institute for Health and Care Excellence (NICE) monitoring committee to review and recommend digital health technologies for children and young people’s mental health.	36.4
Digital health technologies for children and young people’s mental health need to be accessible to all children and young people, including those with communication barriers.	36.4
There should be a list of NICE/NHS recommended digital health technologies for children and young people’s mental health.	36.4
It would be beneficial for organisations delivering children and young people’s mental health support to appoint a digital lead or digital champion to support the implementation of new digital health technologies into their services.	36.4

## 4 Discussion

### 4.1 Summary of findings

Increasing the use of digital therapeutic interventions within children and young people’s mental health services has the potential to play an important role in improving access to evidence-based treatment. For this potential to be realised, it is vital to understand how these technologies are viewed by the full range of stakeholders on whom their successful adoption and sustainment depends. We conducted a series of interrelated studies to gain insights into the use of digital therapeutic interventions within NHS-funded children and young people’s mental health services from the perspectives of multiple stakeholders. This included seeking the views of young people and their families, healthcare professionals, service managers and commissioners, policy leads, academics, and technology developers.

The first study aimed to explore the views of digital therapeutic interventions of young people and their parents/carers, with a particular focus on families who face additional barriers to accessing mental health support from NHS services. The findings revealed considerable enthusiasm for the digital intervention approaches and few perceived barriers to their use by young people and their families. Parents/carers emphasised the need to balance engagement with effectiveness and the desire for more guidance with an apparent need to ensure a flexible approach to accommodate varied delivery preferences.

There was a contrast in the views of young people and parents/carers regarding how much structure should be imposed one their use of digital interventions, with parent/carers wanting clear protocols and young people expressing a desire for a more ‘on-demand’ approach. A successful digital offer must balance the needs of these two stakeholders: it will be important to respect the needs of the young person for autonomy and agency by allowing them a degree of choice and privacy in when and where a digital intervention is used, while providing a framework that reassures parents/carers that their usage is safe and aligned with protocols for optimal outcomes. This could, for example, take the form of built-in thresholds for the total time spent engaging with a particular aspect, or tools for parents/carers to monitor and support their child’s interaction with the intervention (while still allowing for a developmentally appropriate levels of privacy).

The second study aimed to understand the views of healthcare professionals, service managers and commissioners on how best to implement and sustain the use of digital therapeutic interventions within children and young people’s mental health services. In common with the families who participated in the first study, professionals participating in Study 2 expressed largely positive views of digital therapeutic interventions. However, unlike among the families, the view that digital interventions are ‘second best’ to traditional face-to-face interventions appeared to be widespread among the professionals. Most professionals viewed digital interventions as being most suitable as waitlist or adjunct interventions but agreed that they were not yet sufficiently embedded within clinical pathways.

Focus group participants identified a need for clearer guidance and leadership regarding the selection and implementation of digital interventions, and dedicated budgets to support sustainable funding. The need to consider how digital therapeutic interventions can both contribute to and help to overcome barriers to access was highlighted, and co-production with young people and their families was agreed to be important.

Study 3 aimed to achieve a consensus across stakeholder groups on the actions needed to bridge the gap between development and implementation of digital therapeutic interventions for children and young people’s mental health. Focus groups with technology professionals and parent/carers yielded several themes that overlapped with the findings of Studies 1 and 2. These included enthusiasm for digital approaches, the importance of aligning digital innovations with service requirements and processes, the need for alternative approaches to evidence generation in the digital space, the need to upskill the mental health workforce, and the importance of co-production with families. These focus groups also highlighted the lack of clear pathways to implementation within the NHS faced by technology developers. The most agreed upon statements emerging from the three online survey rounds encompassed the need for: a) co-production with children and parents/carers, b) enhanced national guidance and local leadership, c) integration of digital offers with existing clinical pathways, and d) efforts to ensure accessibility and inclusivity.

Taken together, our findings suggest that there is much current consensus across mental health professionals, commissioners, academics, policy leads, industry experts, parent/carers and young people regarding the potential benefits of digital therapeutic interventions within children and young people’s mental health services. Further, there is also a high degree of agreement regarding actions needed for these potential benefits to be realised.

### 4.2 Implications and recommendations

The studies we conducted are a contribution to a wider movement towards understanding how the potential benefits of digital technology can be harnessed to support young people’s mental health effectively. Collaborating with children and young people and their parents and carers when designing and implementing mental health services is increasingly recognised as vital (20), and the value of this in relation to digital interventions in particular has been previously noted (21).

The high value placed on co-production across stakeholder groups is reflected in their high ranking in our consensus exercise. However, the findings of the focus groups we conducted suggests that, despite the high value placed on it, co-production is not currently a consistent feature of the development, commissioning or implementation of digital technologies for children and young people’s mental health. Therefore, there is a need for clear expectations regarding co-production approaches to be integrated within national strategy and guidance. Our project’s young advisors recommended that young people should be involved as leaders in the creation and implementation of a national strategy for co-production of digital mental health interventions for children and young people.

The need for clarification regarding the evidence that is sufficient for digital mental health interventions to be recommended to the public we identified has also been recognised in previous work. A study by Batterham and colleagues (22) gained stakeholder perspectives on this issue and found consensus on the need for high-quality evidence of effectiveness, safety and acceptability; however, there was a lack of consensus regarding what would constitute sufficiently high-quality evidence of effectiveness for digital therapeutic interventions. This finding was mirrored in the current research, with stakeholders holding varying views about the threshold of evidence they would require to embed digital therapeutic interventions into routine practice.

This consensus aligns with recent policy developments in relation to digital therapeutic interventions. In 2018, NHS England commissioned NICE to develop an evidence standards framework for digital health technologies (23). The framework provides guidance for evaluators, commissioners and technology developers on assessing a digital technology’s performance, economic impact, design factors and deployment considerations. It comprises 21 standards, ranging from complying with existing safety, quality and information governance requirements, to user group research and credibility with professionals. The framework recommends gathering both evidence of effectiveness and evidence that the technology can be successfully deployed in real-world settings. Further guidance has been produced by the Accelerated Access Collaborative with the aim of streamlining the adoption of healthcare innovations within the NHS and supporting technology developers to navigate this process (24).

NICE has recently begun piloting a programme of Early Value Assessments (EVAs) to accelerate the adoption of promising new technologies with the potential to address important unmet needs, whilst encouraging continued evidence generation regarding their impact. One EVA focusing on guided self-help digital cognitive behavioural therapy for children and young people with mild to moderate symptoms of anxiety or low mood provisionally recommended four digital interventions. While NICE is clear that the recommendation of these interventions is time-limited and conditional on further evidence generation, it is currently unclear exactly what evidence would be sufficient for continued endorsement.

All three of our studies highlighted, from the perspectives of various stakeholders, the need to ensure that digital health technologies are integrated within existing mental health care pathways. However, it remains unclear where within clinical pathways it would be appropriate to embed the use of digital interventions. NICE’s EVA of digital cognitive behavioural therapy for children and young people only considered these interventions for use as waitlist or initial treatment options to meet unmet need.

Our young advisors expressed the view that offering digital interventions solely as waitlist or initial treatment options had the potential to reduce engagement by implying it is merely a stopgap as opposed to a potentially effective treatment option. Young advisors suggested that presenting digital interventions as part of a menu of treatment options for young people to choose from would be likely to promote better engagement and, therefore, outcomes. However, further research is needed to clarify whether this approach would be empirically supported. It will also be important to consider how the situation of digital interventions within clinical pathways might impact timeliness and equality of access.

The importance of efforts to ensure equitable access to digital interventions across the diversity of young people and their families was also an important message across the three studies conducted. This is particularly the case for young people with more complex mental, neurodevelopmental or physical health needs. This was also highlighted by a recent review (21), which suggested involving representative end users in the design and development of interventions as a valuable means of improving inclusivity. The NICE EVA of digital cognitive behavioural therapy for children and young people noted the lack of evidence regarding the use of digital interventions with neurodivergent young people, recommending this as an area for future research.

Key recommendations drawn from the findings of the current studies are summarised in **Box 1** below.

Box 1 **Recommendations for optimising the use of digital therapeutic interventions for children and young people’s mental health by NHS-funded services.**

- A national strategy for involving young people and parents/carers in decisions regarding the commissioning and implementation of digital technologies for children and young people’s mental health should be developed in collaboration with young people and parents/carers.
- Accessible, consolidated national guidance should be produced to support decision making regarding the commissioning of digital technologies for children and young people’s mental health by NHS-funded services.
- Guidance for local teams on how to successfully implement and sustain digital therapeutic interventions, including recommendations for the role of digital champions and upskilling staff, should be made widely available.
- Corresponding structured guidance for families to help support the use of digital interventions in the home to best meet the needs of the young person should also be developed.
- Further research and consultation should be carried out to determine the most appropriate way in which to integrate digital therapeutic interventions within existing care pathways.
- The impact of digital therapeutic interventions on equitable access to care and young people’s health inequalities should be closely monitored and further research carried out to determine how these interventions can be made as accessible and inclusive as possible.

### 4.3 Limitations

The studies were conducted in England and focused on NHS-funded services for children and young people. This may limit the transferability of findings to other geographic regions and healthcare systems.

While efforts were made to ensure recruitment across a broad range of stakeholders, all samples were ultimately self-selected. We were reliant on families and professionals volunteering their time to participate in the research, and on organisations agreeing for their staff to be involved. As such, it is likely that the views of participants are not wholly representative of the wider population from which they were drawn. For instance, it is plausible that those who volunteer to participate in a study of digital therapeutic interventions are more likely to be more enthusiastic about their use than the wider public.

We experienced difficulty recruiting families to take part in an interview for Study 1, leading to the addition of the option to respond via an online survey. Whilst this broadened the range of views we were able to gather, this came at the price of reduced depth of responses, limiting the analysis we were able to conduct. There was also a limited range of barriers to accessing support with which participants self-identified, potentially limiting the transferability of the findings to those experiencing other barriers.

Further, only one of the 13 families who participated in Study 1 had experience of using a digital therapeutic intervention for their child’s mental health prior to data collection. We had hoped to recruit more families with direct experience of digital therapeutic interventions via our links with clinical services offering such interventions. However, due to ethical considerations, the research team were unable to identify and contact families who had been offered digital interventions directly. Instead, we were reliant on extremely busy clinical teams to identify and approach potentially eligible families on our behalf, which proved a barrier to recruitment.

As a result, most data collected in Study 1 reflected the general perceptions and attitudes of families towards this care model as a potential offer, rather than their lived experience of using digital interventions. While this information is valuable in understanding the likely acceptability of digital approaches to families who may be offered these interventions in future, it would be beneficial for future research to explore the experiences of a range of families with experience of using digital therapeutic interventions. Similarly, the extent of the experience of digital interventions of the professionals who took part was varied, with some having limited personal experience of offering digital interventions. Further, in Study 3, only parents and carers who had been part of the initial focus groups were invited to take part in the Delphi survey, resulting in a relatively small number of parent/carer respondents relative to professionals.

### 4.4 Conclusion

The findings of the three studies reported in this article suggest that there is consensus across stakeholder groups regarding the potential for digital therapeutic interventions to play an important role in enabling more young people to access timely mental health support. However, it is also evident that a clear national strategy for commissioning and implementation (incorporating meaningful co-production), accessible guidance regarding commissioning decisions, dedicated and sustained funding, and ongoing research and evaluation is needed for this potential to be realised.

## Data Availability

The raw data supporting the conclusions of this article will be made available by the authors, without undue reservation.
