# Supplementary material for: Understanding stakeholder views of the use of digital therapeutic interventions within children and young people’s mental health services
Source: Front Psychiatry. 2025 Feb 11;16:1505345. doi: 10.3389/fpsyt.2025.1505345 (PMC11850353; doi:10.3389/fpsyt.2025.1505345)
Supplement: Supplementary file 1 [file DataSheet1.pdf]

Supplementary Material

1 Supplementary Data

Supplement 1 – List of statements included in first survey round of consensus exercise

1. It would be beneficial for organisations delivering children and young people's mental health (CYP MH) support to appoint a digital lead or digital champion to support the implementation of new digital health technologies into their services.

2. The CYP MH workforce does not have the time to review the safety and effectiveness of new digital health technologies.

3. It is hard for the CYP MH workforce to know which digital health technologies to select based on their quality, safety and effectiveness.

4. The CYP MH workforce does not have the time to implement new digital health technologies into their services.

5. There is a reluctance amongst the CYP MH workforce to engage with new digital health technologies even once they have been implemented into a CYP MH service.

6. A national strategy for digital health technologies for CYP MH needs to be established and made publicly available.

7. There is a lack of understanding about digital health technologies for CYP MH within the NHS.

8. There is not enough guidance to help people choose digital health technologies for CYP MH.

9. When developing digital health technologies for CYP MH a panel of young advisors should be consulted to inform development

10. When developing digital health technologies for CYP MH a panel of parents and carers should be consulted to inform development.

11. There needs to be more research to understand what children and young people want to see from digital health technologies.

12. There is no standardised approach for the implementation of digital health technologies for CYP MH.

13. There is no clear guidance to support developers of digital health technologies for CYP MH to be implemented by the NHS.

14. There should be a National Institute for Health and Care Excellence (NICE) monitoring committee to review and recommend digital health technologies for CYP MH.

15. It is hard for tech developers to understand the different CYP MH systems, providers and pathways.

16. There is a lack of clear guidance available to tech developers trying to implement digital health technologies for CYP MH into the NHS.

17. Organisations delivering CYP MH support need to work together to allow for the implementation of standardised digital health technologies for CYP MH.

18. Currently there is not enough training readily available to the CYP MH workforce to support them to implement new CYP MH digital health technologies.

19. There is a lack of funding for implementation of digital health technologies for CYP MH.

20. Compatibility with NHS IT systems is a significant barrier to implementing new digital health technologies.

21. There is often resistance from NHS IT departments when it comes to the implementation of digital health technologies.

22. It is not currently clear what level of evidence is required to show that a digital health technology is safe and effective for CYP.

23. There is a lack of motivation amongst the CYP MH workforce to engage with new digital health technologies.

24. There is a lack of confidence amongst the CYP MH workforce to engage with new digital health technologies.

25. There should be a list of NICE/NHS recommended digital health technologies for CYP MH.

26. There needs to be clearer guidance on the standard of evidence required for digital health technologies to be considered for implementation into the NHS.

27. There needs to be more information on digital health technologies available to parents and carers so they can make informed decisions.

28. Some organisations delivering CYP MH support do not have the infrastructure to implement digital health technologies.

29. There is no consistent threshold of acceptability of digital health technologies amongst organisations delivering CYP MH support.

30. Commissioners, service managers and the CYP MH workforce need to be better equipped to understand the depth and breadth of digital health technologies for CYP MH.

31. Competencies related to the use and implementation of digital health technologies should be mandatory, and regularly reviewed for the CYP MH workforce.

32. Digital health technologies should be used as preventative treatments for CYP MH.

33. Digital health technologies should be a distinctive pathway for mild/moderate CYP MH concerns.

34. Digital health technologies should be a distinctive pathway for moderate/severe CYP MH concerns.

35. Digital health technologies should be integrated with existing pathways for CYP MH.

36. Digital health technologies for CYP MH should not be offered to children and young people unless they are already receiving face-to-face support from NHS mental health services.

37. Digital health technologies for CYP MH should be offered to children and young people who are on a waiting list for NHS mental health services.

38. Implementation of digital health technologies within organisations delivering CYP MH support is a whole team responsibility and should be built into everyone's job descriptions.

39. There needs to be a digital lead within organisations delivering CYP MH support that is able to support other staff to use digital health technologies.

40. Decisions to adopt and implement digital tools should be based on NICE guidelines.

41. Developers of digital health technologies for CYP MH should provide implementation training to the CYP MH workforce.

42. It is not the responsibility of developers of digital health technologies for CYP MH to provide implementation training to the CYP MH workforce.

43. Parents and carers need guidance and support on how to manage safety and risks for their children accessing digital health technologies for CYP MH.

44. There is a lack of guidance about which digital health technologies for CYP MH are endorsed by the NHS.

45. There is a lack of guidance about how to assess the quality and safety of digital health technologies for CYP MH.

Supplement 2 – List of statements included in second survey round of consensus exercise.

1. It would be beneficial for organisations delivering children and young people's mental health (CYP MH) support to appoint a digital lead or digital champion to support the implementation of new digital health technologies into their services.

2. It is hard for the CYP MH workforce to know which digital health technologies to select based on their quality, safety and effectiveness.

3. A national strategy for digital health technologies for CYP MH needs to be established and made publicly available.

4. There is not enough guidance to help people choose digital health technologies for CYP MH.

5. When developing digital health technologies for CYP MH a panel of young advisors should be consulted to inform development

6. When developing digital health technologies for CYP MH a panel of parents and carers should be consulted to inform development.

7. There needs to be more research to understand what children and young people want to see from digital health technologies.

8. There should be a National Institute for Health and Care Excellence (NICE) monitoring committee to review and recommend digital health technologies for CYP MH.

9. Organisations delivering CYP MH support need to work together to allow for the implementation of standardised digital health technologies for CYP MH.

10. Currently there is not enough training readily available to the CYP MH workforce to support them to implement new CYP MH digital health technologies.

11. There should be a list of NICE/NHS recommended digital health technologies for CYP MH.

12. There needs to be clearer guidance on the standard of evidence required for digital health technologies to be considered for implementation into the NHS.

13. There needs to be more information on digital health technologies available to parents and carers so they can make informed decisions.

14. Commissioners, service managers and the CYP MH workforce need to be better equipped to understand the depth and breadth of digital health technologies for CYP MH.

15. Digital health technologies should be integrated with existing pathways for CYP MH.

16. Digital health technologies for CYP MH should be offered to children and young people who are on a waiting list for NHS mental health services.

17. There needs to be a digital lead within organisations delivering CYP MH support that is able to support other staff to use digital health technologies.

18. Decisions to adopt and implement digital tools should be based on NICE guidelines.

19. Developers of digital health technologies for CYP MH should provide implementation training to the CYP MH workforce.

20. Parents and carers need guidance and support on how to manage safety and risks for their children accessing digital health technologies for CYP MH.

21. There is a lack of guidance about which digital health technologies for CYP MH are endorsed by the NHS.

22. There is a lack of guidance about how to assess the quality and safety of digital health technologies for CYP MH.

23. Children and young people accessing mental health support should be offered the choice of digital health technologies or face to face appointments.

24. When children and young people's access digital health technologies for mental health, this should be counted towards access targets for NHS CYP MH services.

25. Digital health technologies for CYP MH need to be accessible to all children and young people, including those with communication barriers.

26. CYP, parents and carers and the CYP MH workforce should contribute to decisions about which digital health technologies should be developed.

27. Where they are involved in the development of digital health technologies, it should be clear to parents, carers, children and young people how their data will be used and stored.

28. There is a lack of clear communication about national guidance and the tools available to those seeking to develop, use or fund digital health technologies for CYP MH.

Supplement 3 – List of statements included in third survey round of consensus exercise.

1. When developing digital health technologies for CYP MH a panel of young advisors should be consulted to inform development.

2. A national strategy for digital health technologies for CYP MH needs to be established and made publicly available.

3. When developing digital health technologies for CYP MH a panel of parents and carers should be consulted to inform development.

4. Digital health technologies should be integrated with existing pathways for CYP MH.

5. There should be a National Institute for Health and Care Excellence (NICE) monitoring committee to review and recommend digital health technologies for CYP MH.

6. Digital health technologies for CYP MH need to be accessible to all children and young people, including those with communication barriers.

7. There should be a list of NICE/NHS recommended digital health technologies for CYP MH.

8. It would be beneficial for organisations delivering children and young people’s mental health (CYP MH) support to appoint a digital lead or digital champion to support the implementation of new digital health technologies into their services.

9. CYP, parents and carers and the CYP MH workforce should contribute to decisions about which digital health technologies should be developed.

10. When children and young people’s access digital health technologies for mental health, this should be counted towards access targets for NHS CYP MH services.

11. There needs to be more research to understand what children and young people want to see from digital health technologies.

12. Commissioners, service managers and the CYP MH workforce need to be better equipped to understand the depth and breadth of digital health technologies for CYP MH.

13. Organisations delivering CYP MH support need to work together to allow for the implementation of standardised digital health technologies for CYP MH.

14. Parents and carers need guidance and support on how to manage safety and risks for their children accessing digital health technologies for CYP MH.

15. There needs to be a digital lead within organisations delivering CYP MH support that is able to support other staff to use digital health technologies.

16. Where they are involved in the development of digital health technologies, it should be clear to parents, carers, children and young people how their data will be used and stored.

17. Developers of digital health technologies for CYP MH should provide implementation training to the CYP MH workforce.
